# Identification of the retinoschisin-binding site on the retinal Na/K-ATPase

**DOI:** 10.1371/journal.pone.0216320

**Published:** 2019-05-02

**Authors:** Karolina Plössl, Kristina Straub, Verena Schmid, Franziska Strunz, Jens Wild, Rainer Merkl, Bernhard H. F. Weber, Ulrike Friedrich

**Affiliations:** 1 Institute of Human Genetics, University of Regensburg, Regensburg, Germany; 2 Institute of Biophysics and Physical Biochemistry, University of Regensburg, Regensburg, Germany; 3 Institute of Clinical Microbiology and Hygiene, University Hospital of Regensburg, Germany, Regensburg, Germany; Medical College of Wisconsin, UNITED STATES

## Abstract

X-linked juvenile retinoschisis (XLRS) is a hereditary retinal dystrophy, caused by mutations in the *RS1* gene which encodes the secreted protein retinoschisin. In recent years, several molecules have been proposed to interact with retinoschisin, including the retinal Na/K-ATPase, L-voltage gated Ca^2+^ channels, and specific sugars. We recently showed that the retinal Na/K-ATPase consisting of subunits ATP1A3 and ATP1B2 is essential for anchoring retinoschisin to plasma membranes and identified the glycosylated ATP1B2 subunit as the direct interaction partner for retinoschisin. We now aimed to precisely map the retinoschisin binding domain(s) in ATP1B2. In general, retinoschisin binding was not affected after selective elimination of individual glycosylation sites *via* site-directed mutagenesis as well as after full enzymatic deglycosylation of ATP1B2. Applying the interface prediction tool PresCont, two putative protein-protein interaction patches (“patch I” and “patch II”) consisting each of four hydrophobic amino acid stretches on the ATP1B2 surface were identified. These were consecutively altered by site-directed mutagenesis. Functional assays with the ATP1B2 patch mutants identified patch II and, specifically, the associated amino acid at position 240 (harboring a threonine in ATP1B2) as crucial for retinoschisin binding to ATP1B2. These and previous results led us to suggest an induced-fit binding mechanism for the interaction between retinoschisin and the Na/K-ATPase, which is dependent on threonine 240 in ATP1B2 allowing the accommodation of hyperflexible retinoschisin spikes by the associated protein-protein interaction patch on ATP1B2.

## Introduction

X-linked juvenile retinoschisis (XLRS, OMIM #312700) is a hereditary retinal dystrophy, which mainly affects males due to its X-linked recessive mode of inheritance and has a world-wide prevalence ranging between 1:5,000 and 1:25,000 [1]. Defects in splitting of retinal layers (“schisis”) and visual signal transmission (“negative ERG”) are characteristic hallmarks of the disease [2].

The *RS1* gene is specifically expressed in photoreceptor and bipolar cells of the retina, as well as in pinealocytes of the pineal gland [3–5]. Mutations in this gene, which encodes retinoschisin, are causative for XLRS [4]. The secreted retinoschisin protein binds to retinal membranes, exhibiting a predominant localization at the inner photoreceptor segments and plexiform layers [6].

Previous studies offer a variety of molecules as possible retinoschisin interaction partners: galactose [7], phosphatidylserine [8, 9], extracellular matrix (ECM) proteins like laminin [10], L-type voltage gated ion channels [11, 12], as well as the retinal Na/K-ATPase [6, 13]. We recently showed that the retinal Na/K-ATPase consisting of the two subunits ATP1A3 (“α3”) and ATP1B2 (“β2”) is responsible for anchoring retinoschisin to retinal membranes [13]. Na/K-ATPases are heterodimeric complexes composed of a single α and a single β subunit and function as an ion pump which is ubiquitously expressed (reviewed by [14, 15]). Four different isoforms of the α subunit and three different isoforms of the β subunit of the Na/K-ATPase have been identified [14] and were shown to be expressed in a tissue specific manner but with unlimited compatibility, i.e. any α subunit can be associated with any β subunit ([16, 17]; reviewed in [14, 15]). The α subunits are integral membrane proteins with 110–130 kDa in size, exhibiting 10 transmembrane domains, 5 short extracellular loops, and 4 cytosolic domains [18]. Overall, 87% amino acid (aa) sequence identity is shared by the α1, α2, and α3 isoforms, while α4 is about 78% identical to the other three α isoforms [19]. The β subunits are single-span transmembrane proteins with a short cytosolic N-terminal part and a large extracellular domain (reviewed in [14, 15]). The three different isoforms of the β subunit show only little aa sequence identity (35–47% in humans) [15]. Their peptide chains are between 31.5 and 35 kDa in size, and posttranslationally modified, particularly by N-linked glycosylation [20].

After co-expression of nine different isozyme combinations of α and β subunits (ATP1A1, ATP1A2, ATP1A3 in combination with ATP1B1, ATP1B2, ATP1B3), we recently showed that ATP1B2 is specifically required for retinoschisin binding, while the α subunits were interchangeable [21]. We now aimed to refine the mapping of the ATP1B2 interaction site with retinoschisin.

## Materials and methods

### Animal model

The *Rs1h*^*-/Y*^ mouse was generated as described earlier [22] and bred onto a C57BL/6 background for more than 10 generations. Mice were housed under specific pathogen-free barrier conditions at the Central Animal Facility of the University of Regensburg and maintained under conditions established by the institution for their use, in strict compliance with NIH guidelines. Mice were sacrificed 14 days after birth by cervical dislocation. Details on institutional animal care and use are provided in **S1 Materials and Methods**.

### Cell culture

HEK293 (human embryonic kidney) cells (“HEK293-EBNA cells”, Invitrogen, Carlsbad, CA, USA) were grown in DMEM high glucose medium containing 10% FCS (fetal calf serum), 100 U/ml penicillin/streptomycin, and 500 μg/ml G418. For the cultivation of HEK293 cells stably expressing recombinant retinoschisin, hygromycin (150 μg/ml) was additionally applied. Y79 cells (human retinoblastoma) (ATCC, Manassas, VA, USA) were cultivated in RPMI medium with 10% FCS as well as 100 U/ml penicillin/streptomycin. Cell lines were grown in a 37°C incubator with a 5% CO_2_ environment and subcultured when they reached 90% confluency for HEK293 cells or a concentration of 4–5 x10^5^ cells/ml for Y79. All media and cell culture supplies were purchased from Life Technologies (Carlsbad, CA, USA).

### Transfection

For transfection of Hek293 cells, the Bio Mirus *Trans*IT-LTI Transfection reagent from Thermo Fisher Scientific (Waltham, MA, USA) was used according to the manufacturer's instructions.

### Antibodies

Primary antibodies against ATP1A3 (#MA3-915) and ATP1B2 (#PA5-26279) were obtained from Thermo Fisher Scientific (Waltham, MA, USA). The ACTB antibody (#A5441) was purchased from Sigma Aldrich, antibodies against ATP1B1 (#15192-1-AP) and ATP1A1 (#55187-1-AP) were obtained from Proteintech (Rosemont, IL, USA). Secondary anti-rabbit or anti-mouse IgG horseradish peroxidase (HRP)-linked antibodies were from Calbiochem (Merck Chemicals GmbH, Schwalbach, Germany). The secondary anti-rabbit Alexa Fluor 488 conjugated antibody used in FACS analysis was obtained from Thermo Fisher Scientific. All commercial antibodies were used to the manufacturer’s recommendations for Western blotting or FACS analysis, respectively. The polyclonal RS1 primary antibody (diluted 1:10000 for Western blot) was kindly provided by Prof. Robert Molday, University of British Columbia, Vancouver, Canada.

### Expression cloning

The bicistronic expression construct was generated by the following consecutive cloning steps: First, the ATP1B2 coding sequence was cloned into pcDNA3 *via* KpnI and XhoI. A partial ATP1B2 expression cassette including a CMV promoter sequence and the ATP1B2 coding sequence was then excised from the pcDNA3 construct *via* NruI and XhoI. The target vector pCEP4 was also digested *via* NruI and XhoI leading to the excision of its hygromycin resistance cassette (3’ of NruI), of one (of two) SalI restriction sites, of the CMV promoter and of the multiple cloning site until XhoI. The partial ATP1B2 expression cassette was ligated into the remaining vector backbone, resulting in a full ATP1B2 expression cassette within a pCEP4 backbone (“pCEPw/oHygro-ATP1B2”). The ATP1A3 coding sequence was cloned into pCEP4 (empty cloning vector) *via* NotI and BamHI. An ATP1A3 expression cassette, consisting of a CMV promoter, the ATP1A3 coding sequence, and a polyadenylation (PolyA) sequence was subsequently excised *via* digestion with SalI and ligated into SalI digested pCEPw/oHygro-ATP1B2, resulting in a bicistronic vector harboring expression cassettes for ATP1B2 and ATP1A3. Primers for generation of ATP1A3 and ATP1B2 coding sequences are given in **S1 Table**.

Expression constructs for ATP1B2 variants lacking different glycosylation sites were generated by site-directed mutagenesis of the full length ATP1B2 coding sequence using Pfu Ultra II Fusion HS DNA Polymerase by Agilent Technologies, Santa Clara, CA, USA and primers given in **S1 Table**.

Expression constructs for RS1-R141H as well as for ATP1B2 patch mutants were generated by site-directed mutagenesis of the respective, non-mutant coding sequences using the Q5 Site-Directed Mutagenesis Kit (New England Biolabs, Ipswich, MA, USA) with primers listed in **S1 Table**. For ATP1B2 patch mutants, assigned hydrophobic peptide sequences of ATP1B2 were exchanged by the corresponding sequences of ATP1B1. To generate the ATP1B2 patch I region 4 mutant, the N-terminal and the C-terminal part of ATP1B2 with overhangs for the additional aa at position 227–237 of ATP1B1 (introducing a ScaI restriction site within the region, without altering its aa sequence) were amplified with primers given in **S1 Table**. N-terminal and C-terminal fragments were then fused together *via* the ScaI restriction site. The ATP1B2 patch II region 2 mutant was generated in three subsequent mutagenesis reactions with three different sets of primers (**S1 Table**).

Comparable transfection and heterologous expression efficiencies of the different ATP1B2 mutants was documented *via* Western blot analysis, followed by densitometric quantification (**S1 Fig**).

### Isolation of cell surface proteins *via* Pierce Cell Surface Protein Isolation Kit (Thermo Fisher Scientific)

HEK293 cells cultivated in 6-well plates were transfected with expression constructs for the different ATP1B2 variants in combination with an ATP1A3 expression construct. 48 h later, cell surface proteins were biotinylated using the Pierce Cell Surface Protein Isolation Kit (Thermo Fisher Scientific) and purified according to the manufacturer’s instructions. Purified membrane proteins were subjected to Western blot analyses.

### FACS analysis

FACS analyses were performed as described in [21]. Briefly, 3x10^5^ Hek293 cells heterologously expressing ATP1A3 and the different ATP1B2 variants were washed twice in 300 μl PBS +1% FCS (5 min, 300xg, 4°C) and then incubated in primary antibody solution (anti-ATP1B2, 1:50, in PBS +1% FCS) for 25 min. After two washing steps (conditions as before), cells were subjected to secondary antibody solution (Alexa-Fluor 488 conjugated anti-rabbit, 1:100, in PBS +1% FCS) for 25 min, followed again by two washing steps (conditions as before). Cells were resuspended in 100 μl PBS +1% FCS and subjected to FACS analysis using a FACSCanto-II flow cytometer run by Diva software (Ver. 7.0, BD Biosciences, San Jose, CA, USA). During the procedure, cells were kept on ice and pre-cooled solutions were applied.

### Enrichment of plasma membranes

Plasma membrane enrichment was performed similar to [13]: A 10 cm dish of HEK293 cells transiently transfected with ATP1B2 and ATP1A3 expression constructs or 4x10^7^ Y79 cells were resuspended in 400 μl TBS. Cells were lysed by sonication. The lysates were then centrifuged at 5,000xg for 4 min at 4°C, the supernatant was transferred to a fresh tube and centrifuged again at 16,000xg for 30 min at 4°C. The supernatant was removed and the pellet resuspended in 400 μl of TBS by sonication. After a final centrifugation step at 16,000xg for 30 min at 4°C the enriched membrane fractions were solubilized in 110 μl TBS by sonication. All steps were carried out on ice or in pre-cooled centrifuges.

### Enzymatic deglycosylation by PNGase F (peptide-N4-(N-acetyl-β-glucosaminyl)asparagine amidase) treatment

Enzymatic removal of N-linked glycan side chains from membrane associated proteins was achieved by treating enriched plasma membrane fractions with PNGase F (New England Biolabs). 25 μl of enriched plasma membrane fraction were incubated with 3 μl of 10X Glycobuffer 2 and 2 μl (1000 U) of PNGase F or control buffer at 37°C for 18 h.

For immunocytochemical analyses, transfected HEK293 cells cultivated in 24-well plates were treated as described above, in a total volume of 100 μl containing 83 μl TBS, 10 μl 10X Glycobuffer 2, and 7 μl (3500 U) of PNGase F or control buffer.

### RS1 binding to Y79 cells, HEK293 cells and *Rs1h*^*-/Y*^ murine retinal explants

Retinoschisin (and RS1-R141H) binding to Y79 cells, *Rs1h*^*-/Y*^ murine tissues or to HEK293 cells heterologously transfected with expression constructs for ATP1A3 and ATP1B2 variants was assessed at least three times in independent assays as described by [13], but with a prolonged incubation time of 1 h.

For testing the binding of recombinant retinoschisin to enzymatically deglycosylated membrane fractions, the same binding assay protocol was applied, however, centrifugation steps were carried out at 16,000xg for 15 min at 4°C.

For binding assays in the presence of increasing amounts of sugars, respective amounts of glucose, galactose and mannose (final concentrations of 0.5 M, 0.75 M and 1 M) were dissolved in retinoschisin containing cell culture supernatant (from HEK293 cells stably transfected with a retinoschisin expression construct). HEK293 cells transfected with expression constructs for ATP1A3 and ATP1B2 were then subjected to binding assays with these supernatants as described above.

### Sodium dodecyl sulfate-polyacrylamide gel electrophoresis (SDS-PAGE) and Western blot analysis

SDS-PAGE, Western blot analyses and densitometric quantification of Western blots were performed as described in [13, 21, 23].

### Statistical analyses

For statistical evaluation of retinoschisin binding and heterologous expression of ATP1B2 mutants the Kruskal Wallis test was used followed by Dunn’s post-test. The calculations were done with the R software (http://www.R-project.org/).

### Immunocytochemistry

Immunocytochemistry with antibodies against retinoschisin (diluted 1:500) and ATP1B2 (diluted 1:1000) was performed as described by Karlstetter and colleagues [24]. The sections were counterstained with 4′,6-diamidino-2-phenylindol (DAPI, 1:1000, Molecular Probes, Leiden, The Netherlands). Images were taken with a fluorescence microscope (Axioskop2 mot plus, Zeiss, equipped with a digital camera) with 63x magnification. Pictures for S3 Fig were taken at 40x magnification and image processing was achieved with the Axiovision software with integrated Z-stack, 3D deconvolution and extended focus modules (Zeiss).

### Identification of putative protein-protein interfaces

A homology model of human ATP1B2 was calculated by using I-TASSER [25] with default parameters and the β subunit structure from *Squalus acanthias* (PDB-ID 3a3y) as a template.

Interfaces were predicted *in silico* by means of PresCont [26]. This program requires as input a protein 3D structure and a multiple sequence alignment (MSA). Here, the ATP1B2 homology model and an MSA of 142 β subunits served as input to identify α subunit interfaces.

### Calculation of solvent accessible surface area

The PyMOL (Schrödinger Inc.) function get_area, with the setting dot_solvent on was used to determine the solvent accessible surface area (SASA) of individual residues. To improve accuracy of the measurement, dot_density was set to the maximum (4); for all other parameters default settings were used. To calculate the accessibility of T240, the specific SASA (13 Å^2^) was compared to the maximal SASA of a threonine, which is 102 Å^2^ [27].

## Results

### The influence of ATP1B2-glycosylation on binding of retinoschisin to the retinal Na/K-ATPase

Dyka and colleagues observed a high affinity of retinoschisin to agarose-coupled galactose [7]. The Na/K-ATPase subunit ATP1B2 which is responsible for retinoschisin binding [21] is a highly glycosylated protein with 8 potential N-glycosylation sites: asparagine (N) 96, N118, N153, N159, N193, N197, N238, and N250 [28]. We therefore speculated that anchorage of retinoschisin to the retinal Na/K-ATPase may be mediated *via* an interaction with the sugar moieties of ATP1B2.

**Fig 1:**
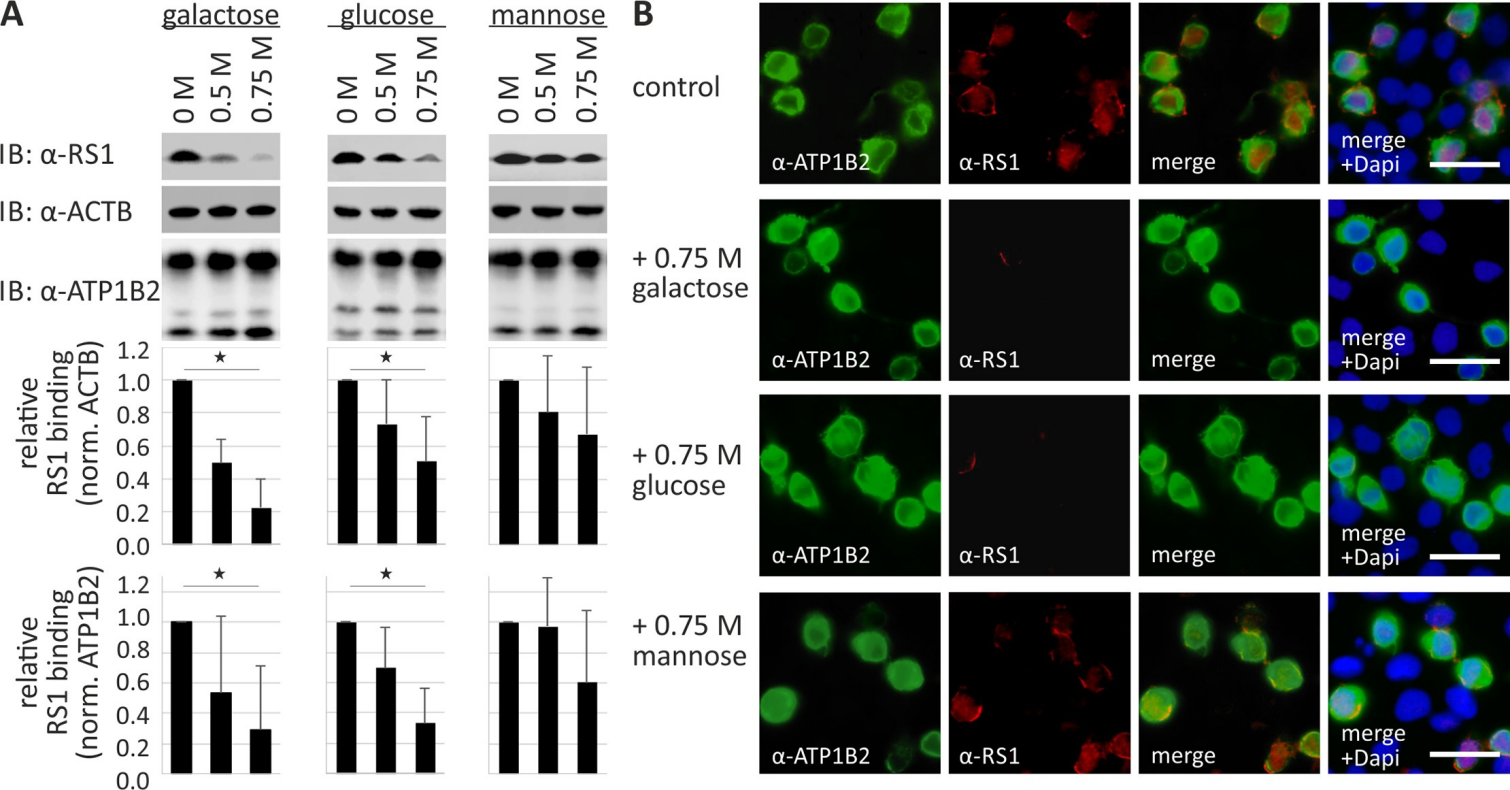
Retinoschisin binding in the presence of galactose, glucose, and mannose. **(A)** HEK293 cells transfected with a bicistronic expression construct for ATP1A3 and ATP1B2 for 48 h were subjected to recombinant retinoschisin for 1 h in the presence of 0, 0.5, or 0.75 M galactose, glucose, or mannose, followed by intensive washing. Retinoschisin binding was investigated by Western blot analyses with antibodies against retinoschisin. ACTB staining served as loading control. Densitometric quantification of retinoschisin binding was performed on immunoblots from 4 individual experiments. Signals were normalized against ACTB and calibrated against signals for 0 M sugar. Data represent the mean + SD. Underlined asterisks mark statistically significant (* = P < 0.05) differences. **(B)** HEK293 cells transfected with a bicistronic expression construct for ATP1A3 and ATP1B2 for 48 h were subjected to recombinant retinoschisin for 1 h in the presence of 0 M (control) or 0.75 M galactose, glucose, or mannose, followed by intensive washing. Subsequently, retinoschisin binding was analyzed *via* immunocytochemistry with antibodies against retinoschisin and ATP1B2. Scale bars, 40 μm.

First, we examined the binding capacity of retinoschisin to the Na/K-ATPase (heterologously expressed in HEK293) in the presence of different sugars solubilized in the incubation medium, assuming a competition of the externally added sugars with the ATP1B2 glycoside side chains for binding to retinoschisin **(Fig 1)**.

We have previously shown that retinoschisin binds to the surface of HEK293 cells heterologously expressing the two subunits of the retinal Na/K-ATPase (ATP1A3 and ATP1B2) while no binding was observed when the two retinal Na/K-ATPase subunits were absent [13, 21, 23]. We have used this assay to transfect HEK293 cells with a bicistronic expression vector for ATP1A3 and ATP1B2 for 48 h and incubated the cells with retinoschisin in the presence of galactose, glucose, or mannose (0, 0.5, and 0.75 M). After intensive washing, retinoschisin binding was assessed by Western blot analysis of cell pellet lysates. We observed decreased binding of retinoschisin in the presence of galactose and glucose (22.4 +/- 17.6% with 0.75 M galactose, and to 50.5 +/- 27.3% with 0.75 M glucose after normalization against ACTB, 29.0 +/- 41.8% with 0.75 M galactose, and to 33.2 +/- 22.8% with 0.75 M glucose after normalization against ATP1B2, p < 0.05, **Fig 1A**). Interestingly, mannose did not exert a similar effect: no statistically significant decrease in binding of retinoschisin to the heterologously expressed Na/K-ATPase was observed in the presence of 0.5 or 0.75 M mannose **(Fig 1A)**. In addition to Western blot analysis, retinoschisin binding to transfected HEK293 cells in dependence of various sugars was also investigated *via* immunocytochemistry. After transient transfection of HEK293 cells with a bicistronic expression vector for subunits ATP1A3 and ATP1B2, the cells were incubated with retinoschisin in the presence of 0.75 M galactose, glucose, or mannose for 1 h **(Fig 1B)** or 7 h **(S2 Fig)**. In control cells (incubated without any sugar), retinoschisin signals (visualized *via* immunocytochemistry against retinoschisin) were detected at the membranes of transfected cells (identified *via* immunocytochemistry against ATP1B2), but not at non-transfected cells (detected by DAPI staining, **Fig 1B, S2 Fig)**. Incubation with 0.75 M mannose had no significant effect on retinoschisin binding to transfected HEK293 cells **(Fig 1B, S2 Fig)**. After incubation with 0.75 M galactose or glucose, however, retinoschisin signals on transfected HEK293 cells were strongly reduced **(Fig 1B, S2 Fig)**.

The different effects of glucose/galactose and mannose indicate that reduced retinoschisin binding is not a consequence of altered osmolality, but rather of a specific interaction between retinoschisin and galactose or glucose.

Considering the high specificity of retinoschisin to ATP1B2, we assessed whether retinoschisin binding to the retinal Na/K-ATPase may require a particular carbohydrate side chain of ATP1B2. We generated glycosylation deficient ATP1B2 variants, in which each one of the 8 glycosylation sites was destroyed by replacing the asparagine (N) residue with a glutamine (Q) according to [28]. All of these variants as well as normal ATP1B2 were heterologously expressed in combination with ATP1A3 in HEK293 and tested for their capacity to bind externally added retinoschisin. The aa exchange N238Q in ATP1B2 served as a negative control as it was previously shown to result in strong misfolding and ER retention of ATP1B2 [28]. The cells were subsequently incubated with recombinant retinoschisin, intensively washed and subjected to Western blot analysis as described [13].

**Fig 2:**
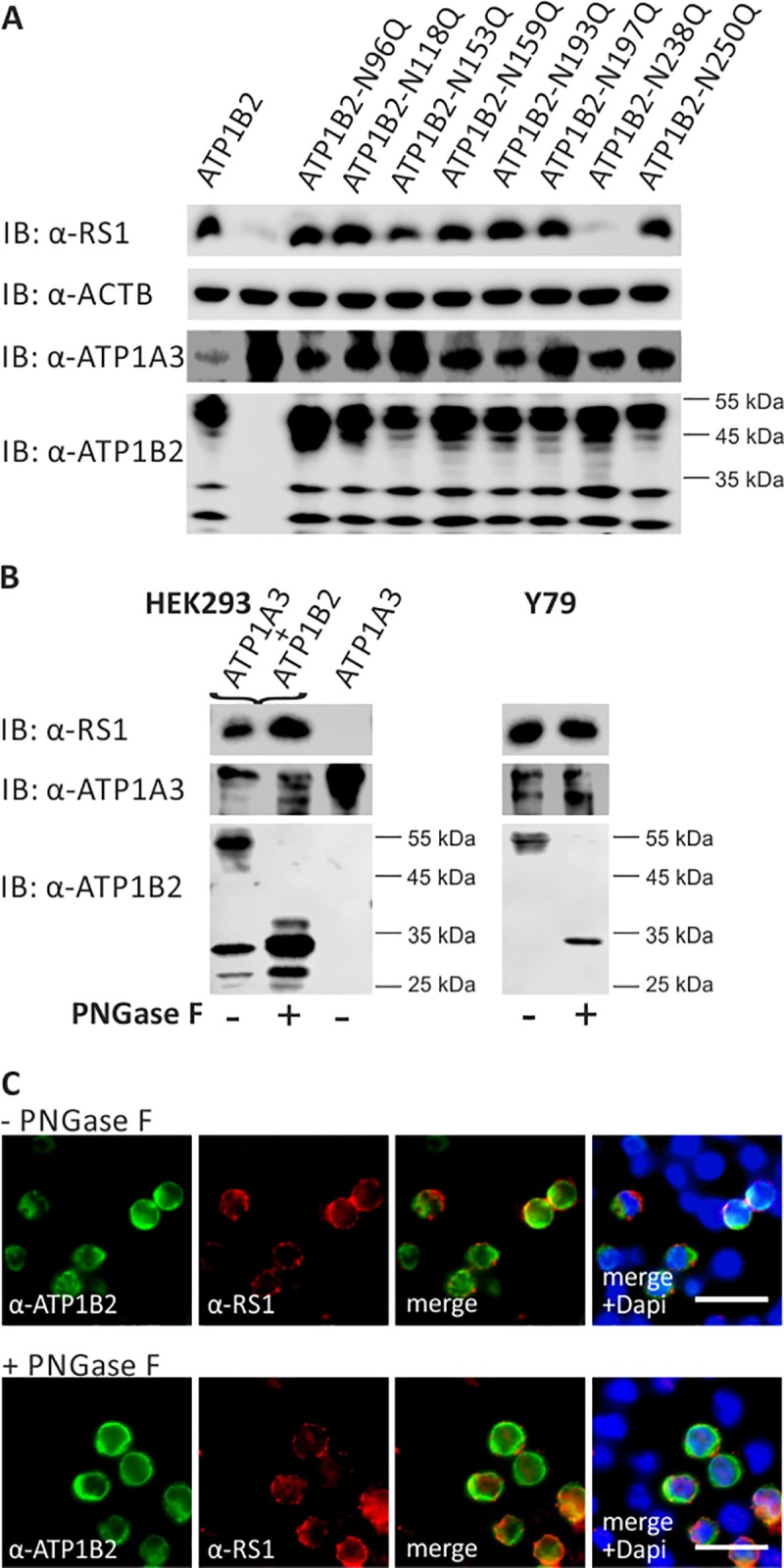
Retinoschisin binding in dependence of ATP1B2 glycosylation. **(A)** HEK293 cells were co-transfected with expression constructs for ATP1A3 and expression constructs for ATP1B2 mutants deficient in each one glycosylation site (ATP1B2-N96Q, -N118Q, -N153Q, -N159Q, -N193Q, -N197Q, -N238Q, and -N250Q). 48 h after transfection, cells were incubated with recombinant retinoschisin for 1 h, followed by intensive washing. Cells transfected with only ATP1A3 expression constructs served as a negative control, cells co-transfected with expression constructs for ATP1A3 and for normal ATP1B2 served as a positive control in the retinoschisin binding assay [13]. Heterologous protein expression as well as retinoschisin binding was investigated by Western blot analyses with antibodies against retinoschisin, ATP1A3, and ATP1B2. The ACTB staining served as loading control. **(B)** Enriched membrane fractions from HEK293 cells transfected with ATP1A3 and ATP1B2 expression constructs and Y79 cells were subjected to enzymatic deglycosylation using PNGase F. A control sample was subjected to the same treatment, but without PNGase F. Subsequently, membrane fractions were incubated with recombinant retinoschisin for 1 h, followed by intensive washing. Retinoschisin binding, (heterologous) Na/K-ATPase expression, and ATP1B2 deglycosylation were investigated by Western blot analyses with antibodies against retinoschisin, ATP1A3, and ATP1B2. Membranes from HEK293 cells transfected with only ATP1A3 expression vectors served as negative control in this retinoschisin binding assay. **(C)** HEK293 cells co-transfected with expression constructs for ATP1A3 and for ATP1B2 for 48 h were subjected to enzymatic deglycosylation using PNGase F as described in **(B)**. A control sample was subjected to the same treatment, but without PNGase F. Subsequently, cells were incubated with recombinant retinoschisin for 1 h, followed by intensive washing. Retinoschisin binding was analyzed *via* immunocytochemistry with antibodies against retinoschisin and ATP1B2. Scale bars, 40 μm.

In Western blot analysis, HEK293 cells heterologously expressing normal ATP1B2 revealed several protein species after anti-ATP1B2 staining **(Fig 2A)**, reflecting different glycosylation intermediates of the ATP1B2 subunit due to elongation and branching by the Golgi-resident glycosidases [28]. The largest protein appeared at a molecular weight of about 55 kDa representing the fully glycosylated, mature species [28]. HEK293 cells heterologously expressing the ATP1B2 glycosylation mutants also exhibited a pattern of protein labeling after anti-ATP1B2 staining. However, the protein species with the highest molecular weight for the different mutants appeared to have a slightly reduced molecular weight compared to normal ATP1B2, likely due to the absence of a single glycoside chain respectively **(Fig 2A)**.

After incubation with recombinant retinoschisin, HEK293 cells heterologously expressing normal ATP1B2 efficiently bound retinoschisin **(Fig 2A)**. Cells heterologously expressing mutant ATP1B2-N238Q which is not transported to the plasma membrane [28], showed no retinoschisin binding **(Fig 2A)**. In contrast, cells transfected with any of the other ATP1B2 glycosylation mutants (ATP1B2-N96, -N118, -N153, -N159, -N193, -N197, or N250) were capable of binding retinoschisin. This result revealed that retinoschisin binding to ATP1B2 does not require any of the individual ATP1B2 glycoside side chains attached to N96, N118, N153, N159, N193, N197, or N250.

Further, we assessed the effect of full ATP1B2 deglycosylation on the capacity of the retinal Na/K-ATPase to interact with retinoschisin. As disruption of multiple glycosylation sites in ATP1B2 causes ER retention of the protein [28], deglycosylation of several or all sites simultaneously *via in vitro* mutagenesis is not applicable. In addition, selective deglycosylation at N238 leads to ER retention [28], making it unfeasible to address an influence of this specific glycosylation site on retinoschisin interaction. We thus applied PNGase F treatment [29] to fully deglycosylate the mature, membrane associated ATP1B2 in transfected HEK293 cells as well as in Y79 cells, an established human retinoblastoma cell line which endogenously expresses the retinal Na/K-ATPase [23]. Subsequently, we performed retinoschisin binding assays on the deglycosylated cells and evaluated the extent of deglycosylation as well as retinoschisin binding *via* Western blot analysis **(Fig 2B)** as well as immunocytochemical analyses **(Fig 2C)**.

In Western blot analyses, HEK293 and Y79 cells not subjected to PNGase F showed labelling for the fully glycosylated, mature ATP1B2 at around 55 kDa. This protein species was no longer present after PNGase F treatment of the cells. Instead, proteins with a size of unglycosylated ATP1B2 (CCDS32550.1; 28.7 kDa) were observed, indicating successful deglycosylation of ATP1B2 **(Fig 2B)**. Deglycosylation, however, did not interfere with retinoschisin binding in either of the tested cell lines **(Fig 2B)**, Similarly, immunocytochemical analyses of HEK293 cells heterologously expressing ATP1A3 and ATP1B2 showed binding of retinoschisin to control treated cells as well as to cells treated with PNGase F **(Fig 2C).** This implies that binding of retinoschisin to the retinal Na/K-ATPase does not require ATP1B2 glycosylation. Another observation additionally supports this conclusion. Dyka and colleagues reported that the retinoschisin mutant RS1-R141H (NM_000330.3(RS1): c.422G>A [p.Arg141His]) exhibit similar binding affinities to galactose as normal retinoschisin [7]. However, a previous study from our group showed that RS1-R141H could not bind to the heterologously expressed retinal Na/K-ATPase and retinal membranes [30]. The latter results were reproduced in this study (**S3 Fig**): Retinoschisin efficiently bound to HEK293 cells heterologously expressing the retinal Na/K-ATPase subunits ATP1A3 and ATP1B2 and to *Rs1h*^*-/Y*^ retinal membranes, while RS1-R141H failed to interact. Neither retinoschisin nor RS1-R141H bound to HEK293 cells not expressing ATP1B2 or to *Rs1h*^*-/Y*^ murine kidney membranes.

Taken together, these experiments indicate that binding of retinoschisin to ATP1B2 is not mediated by an interaction of retinoschisin with glycoside side chains of ATP1B2. The observed sugar affinity of retinoschisin (and RS1-R141H [7]) is thus not related to its anchorage to retinal membranes.

### Predicting regions of protein-protein interaction by bioinformatics tools

Excluding ATP1B2 glycoside side chains as a potential retinoschisin binding component of ATP1B2, we assessed whether retinoschisin might directly interact with a specific ATP1B2 protein surface structure. We used *in silico* prediction to identify putative protein-protein interaction regions on the surface of ATP1B2. As the 3D structure of human ATP1B2 is unknown, we generated a homology model by means of the I-TASSER software suite [25] and utilized the β subunit structure from *S*. *acanthias* (PDB-ID 3a3y) as a template. The C-score of the resulting model was 0.93, which accounts for high quality, and the superposition of the homology model and the template confirmed high structural similarity with a root-mean-square deviation of not more than 0.98 Å.

**Fig 3:**
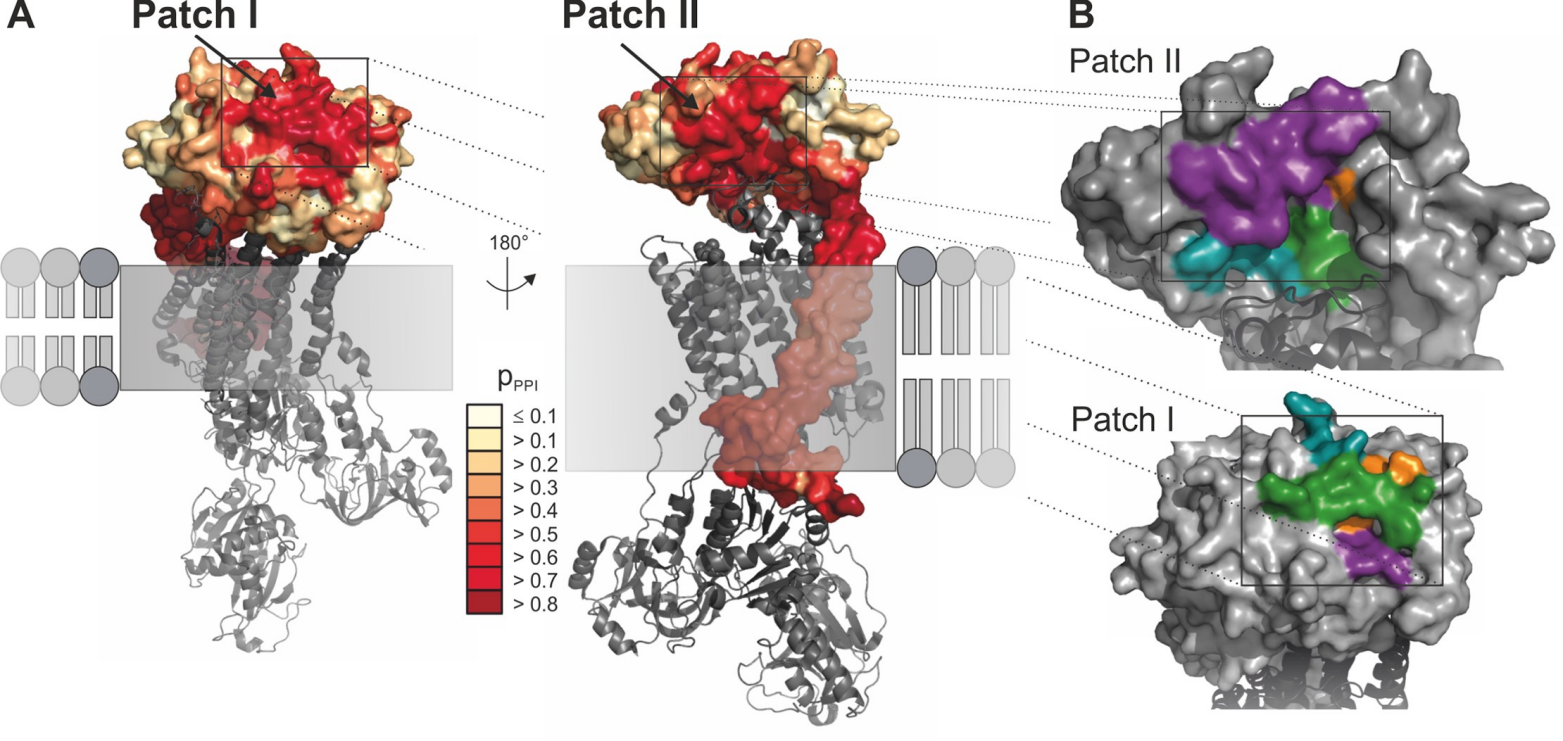
Localization and composition of putative protein-protein interaction sites. **(A)** The putative protein-protein interaction sites identified by PresCont for a homology model of ATP1B2 are colored according to the p_PPI_ score, which is for each residue the probability that it contributes to a protein-protein interaction site. The membrane-bound domain of ATP1B2 is composed of hydrophobic residues, which causes false positive predictions. The localization of the α subunit of the Na/K-ATPase complex from *S*. *acanthias* (PDB-ID 3a3y, in grey) results from a superposition of the corresponding β subunits. **(B)** Detailed view of patch I (lower panel) and II (upper panel). The four regions forming the patch are colored in cyan (region 1), purple (region 2), green (region 3), and orange (region 4). The position of the α subunit colored in dark grey was transferred from the Na/K-ATPase complex of *S*. *acanthias* (PDB-ID 3a3y) by means of superposition.

To predict putative protein interfaces, the homology model and an MSA consisting of 142 β subunit sequences was analyzed by means of PresCont [26]. This algorithm was trained on soluble protein complexes and assesses features like hydrophobicity and sequence conservation to determine for each surface residue the probability of being part of an interface. In addition to the membrane-intruding surface of ATP1B2, which is an artifact due to PresCont’s field of application, two putative protein-protein interaction regions were identified, which we termed "patch I" and "patch II" **(Fig 3A)**. Each patch is composed of four hydrophobic peptides **(Fig 3B)**, termed “region 1” (depicted in cyan), “region 2” (purple), “region 3” (green), and “region 4” (orange), which are specified in **Table 1**.

**Table 1:** Amino acid (aa) positions and sequences of the each four regions building the predicted protein-protein-interfaces patch I and patch II of ATP1B2 as well as corresponding aa sequences in ATP1B1.

ATP1B2 –putative interfaces				ATP1B1
name		aa positions	aa sequence	corresponding aa sequence
Patch I	Region 1	158–163	GNCSGI	GNCSGL
	Region 2	198–201	VTCA	VQCT
	Region 3	246–249	VKFL	VQFT
	Region 4	213–221	NFVMFPANG	NVEYFGLGNSP
Patch II	Region 1	83–88	MIRPKT	TQIPQI
	Region 2	108–121	QKLNKFLEPYNDSI	LNIVRFLEKYKDSA
	Region 3	181–184	KMNR	KLNR
	Region 4	240	T	L

To assess an interaction of retinoschisin with either of the two predicted patches, we altered the composition of patch I and II by applying site-directed mutagenesis. More specifically, ATP1B2 residues belonging to regions 1–4 were replaced by the corresponding ones from ATP1B1 (**Table 1**), which does not interact with retinoschisin [21].

### Retinoschisin binding to ATP1B2 patch I mutants

**Fig 4:**
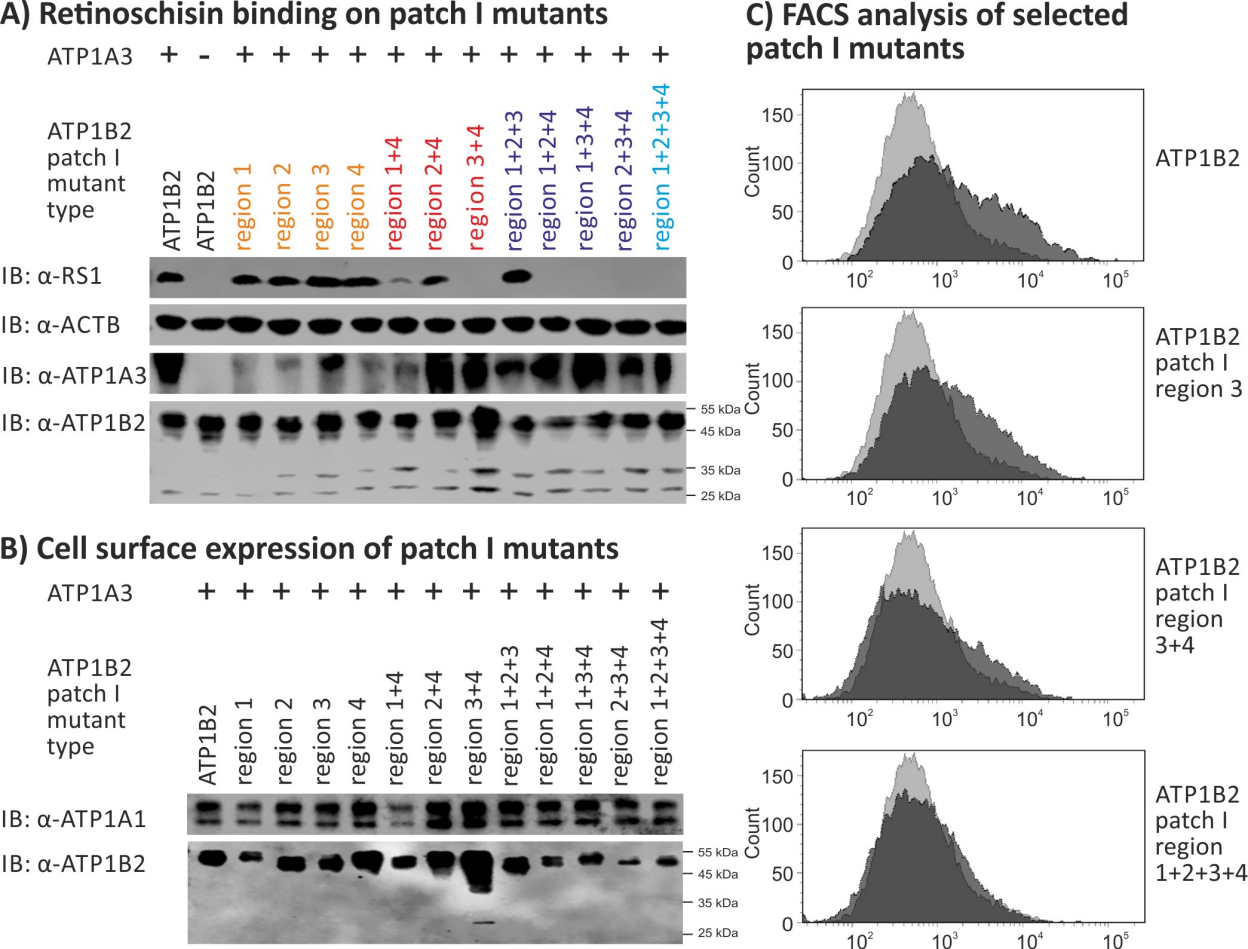
Retinoschisin binding to ATP1B2 patch I mutants. HEK293 cells were transfected with expression constructs for ATP1A3 in combination with expression constructs for different ATP1B2 variants mutated in patch I regions depicted within the figure. **(A)** 48 h after transfection, cells were subjected to recombinant retinoschisin for 1 h, followed by intensive washing. Cells transfected with expression constructs for only ATP1B2 served as a negative control, cells transfected with expression constructs for ATP1A3 and normal ATP1B2 served as a positive control in the retinoschisin binding assay [13]. Heterologous protein expression as well as retinoschisin binding was investigated by Western blot analyses with antibodies against retinoschisin, ATP1A3, and ATP1B2. The ACTB staining served as loading control. **(B)** 48 h after transfection, cell surface proteins were biotinylated, purified by streptavidin affinity chromatography and analysed by Western blotting using antibodies against ATP1B2. The ATP1A1 immunoblot was performed as loading control. **(C)** 48 h after transfection, cells were subjected to FACS analyses applying an anti-ATP1B2 antibody. Representative histograms of cells transfected with selected ATP1B2 patch I mutants are given in this figure. Light grey: histogram of untransfected HEK293 cells depicting unspecific background signals. Dark grey: histogram of transfected HEK293 cells.

Patch I, localized at the top membrane-distal surface of the extracellular domain of ATP1B2, consists of 23 residues and is relatively large with a SASA of 1345 Å^2^. To investigate experimentally an involvement of ATP1B2 patch I in retinoschisin binding, HEK293 cells were transfected with expression constructs for ATP1A3 in combination with expression constructs for different ATP1B2 patch I mutants or normal ATP1B2. The cells were subsequently incubated with externally added recombinant retinoschisin, intensively washed, and tested for retinoschisin binding by Western blot analysis according to [13, 21] **(Fig 4A)**.

All patch I mutants displayed robust heterologous expression in HEK293 cells **(Fig 4A, S1 Fig)** demonstrating that the induced mutations did not interfere with protein synthesis. We observed efficient retinoschisin binding to all ATP1B2 mutants altered in only one of the four hydrophobic regions of patch I (“patch I region 1, region 2, region 3 or region 4” labeled orange in **Fig 4A**). An ATP1B2 patch I mutant, where all of the four regions (“patch I region 1+2+3+4”, bright blue in **Fig 4A**) were altered simultaneously, was no longer able to bind retinoschisin. However, retinoschisin bound to triple mutant ATP1B2 patch I mutated in regions 1+2+3, but not to triple mutants involving region 4 (“patch I region 1+2+4, region 1+3+4, or region 2+3+4”, labeled purple in **Fig 4A**). To further test a putative role of region 4 in retinoschisin binding, double mutants of patch I region 4 combined with either region 1, 2 or 3 (labeled red in **Fig 4**) were generated. Retinoschisin bound to ATP1B2 subunits mutated in patch I regions 2+4 as well as in patch I region 1+4, but not to ATP1B2 mutated in patch I region 3+4 **(Fig 4A)**.

As previous analyses showed negative effects of specific ATP1B2 mutations on membrane transport and outer domain folding [21, 28], we pursued heterologous cell surface expression **(Fig 4B)** and protein folding **(Fig 4C, Table 2)** of the ATP1B2 mutants. Applying cell surface biotinylation with subsequent purification of biotinylated proteins, all ATP1B2 patch I mutants were successfully detected in the respective cell surface protein fraction **(Fig 4B)**, documenting plasma membrane export of all patch I mutants. Of note, the purified cell surface protein fractions only contained the mature, membrane bound ATP1B2 variants with a molecular weight of about 55 kDa (**Fig 4B**). In FACS analyses with anti-ATP1B2 antibodies, however, striking differences in the detection of the different variants were observed: With increasing mutation numbers, ATP1B2-antibody binding by the ATP1B2 patch I mutants strongly decreased, as indicated by a decrease in mean fluorescence intensity compared to non-mutant ATP1B2 (**Table 2, Fig 4C)**. As all ATP1B2 mutants were detected on the cell surface after surface protein isolation (**Fig 4B**), the decreased affinity of the ATP1B2-antibody for its epitope (aa 115 to 141, distinct from patch I) suggests an alteration in the overall 3-dimensional structure of the respective ATP1B2 mutants (similar to results presented in [21, 31, 32]). It is thus conceivable that the overall structural alteration, causing a reduced accessibility of the actual retinoschisin binding site (different from patch I), and not the specific change in patch I is responsible for abolished retinoschisin binding.

**Table 2:** Quantitative FACS analysis of ATP1B2 patch I mutants. Mean fluorescence intensity (MFI) from total cell population, calibrated against non-mutant ATP1B2. Given is the mean +/- SD of 3 independent experiments. Neg. control: untransfected cells.

X-fold mutant type	ATP1B2 patch I variant	MFI (% of non-mutant ATP1B2)	
		Per each ATP1B2 patch I variant	Per ATP1B2 patch I x-fold mutant type
-	Neg. control	32.8 +/- 6.7	32.8 +/- 6.7
Not mutated	ATP1B2	100 +/- 0.0	100 +/- 0.0
Single mutants	Patch I region 1	187.9 +/- 129.6	95.1 +/- 53.0
	Patch I region 2	70.2 +/-22.2	
	Patch I region 3	62.7 +/- 20.1	
	Patch I region 4	60.8 +/- 29.3	
Double mutants	Patch I region 1+4	35.2 +/- 14.4	43.7 +/- 7.4
	Patch I region 2+4	48.8 +/- 14.9	
	Patch I region 3+4	47.1 +/-10.6	
Triple mutants	Patch I region 1+2+3	34.2 +/- 11.7	36.7 +/- 4.4
	Patch I region 1+2+4	37.1 +/- 9.7	
	Patch I region 1+3+4	42.8 +/- 25.8	
	Patch I region 2+3+4	32.7 +/- 13.7	
Quadruple mutant	Patch I region 1+2+3+4	31.9 +/- 7.0	31.9 +/- 7.0

### Retinoschisin binding to ATP1B2 patch II mutants

**Fig 5:**
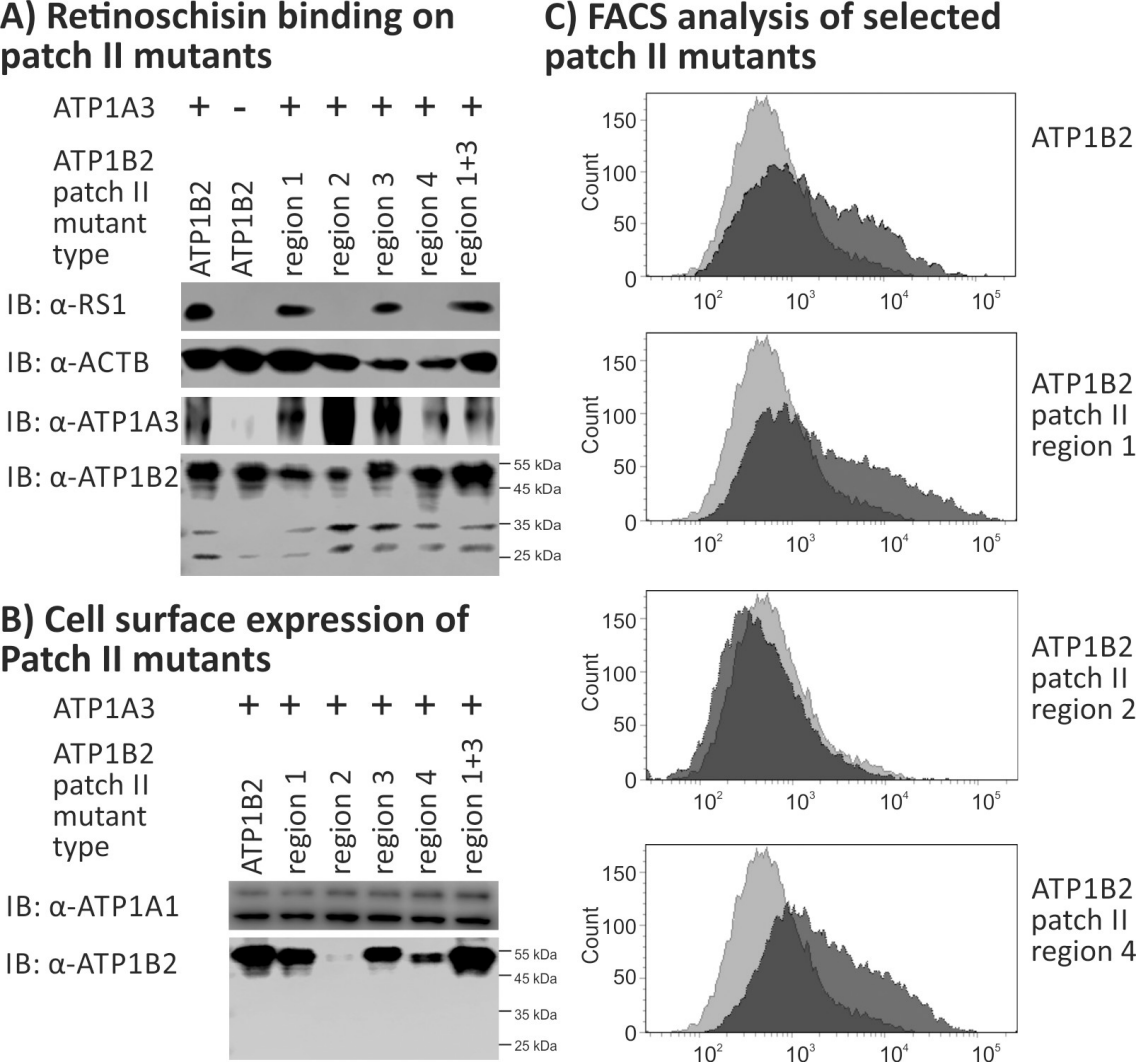
Retinoschisin binding to ATP1B2 patch II mutants. HEK293 cells were transfected with expression constructs for ATP1A3 in combination with expression constructs for different ATP1B2 variants mutated in patch II regions depicted within the figure. **(A)** 48 h after transfection, cells were subjected to recombinant retinoschisin for 1 h, followed by intensive washing. Cells transfected with expression constructs for only ATP1B2 served as a negative control, cells transfected with expression constructs for ATP1A3 and normal ATP1B2 served as a positive control in the retinoschisin binding assay [13]. Heterologous protein expression as well as retinoschisin binding was investigated by Western blot analyses with antibodies against retinoschisin, ATP1A3, and ATP1B2. The ACTB staining served as loading control. **(B)** 48 h after transfection, cell surface proteins were biotinylated, purified by streptavidin affinity chromatography and analysed by Western blotting using antibodies against ATP1B2. The ATP1A1 immunoblot was performed as loading control. **(C)** 48 h after transfection, cells were subjected to FACS analyses applying an anti-ATP1B2 antibody. Representative histograms of cells transfected with selected ATP1B2 patch II mutants are given in this figure. Light grey: histogram of untransfected HEK293 cells depicting unspecific background signals. Dark grey: histogram of transfected HEK293 cells.

Patch II is localized at the lateral surface of ATP1B2, consists of 25 residues and has a SASA of 1214 Å^2^. Similar to our approach for patch I, we initially assessed retinoschisin binding to ATP1B2 patch II mutants by replacing individual regions with the corresponding sequences from ATP1B1 (**Fig 5A**). Similar to patch I mutants, patch II mutants showed robust heterologous expression in HEK293 cells **(Fig 5A, S1 Fig).** ATP1B2 mutated in patch II region 1 or 3 efficiently bound retinoschisin. In contrast, ATP1B2 mutated in patch II region 2 or 4 failed to interact with retinoschisin. A double mutant of patch II regions 1+3 also effectively bound retinoschisin (**Fig 5A**).

Cell surface protein isolation of patch II mutants revealed plasma membrane localization of all ATP1B2 patch II mutants, except of ATP1B2 patch II region 2 mutant **(Fig 5B)**. Consistent with this, FACS results showed anti-ATP1B2 antibody binding to all ATP1B2 patch II mutants, except of ATP1B2 mutated in patch II region 2 **(Table 3, Fig 5C).** The membrane absence of the ATP1B2 patch II region 2 mutant might be a consequence of misfolding with subsequent ER retention as previously observed for other ATP1B2 mutants [28]. The retinoschisin binding incapacity of cells heterologously expressing ATP1B2 patch II region 2 mutant can consequently be explained by the absence of this mutant in the membrane.

**Table 3:** Quantitative FACS analysis of ATP1B2 patch II mutants. Mean fluorescence intensity (MFI) from total cell population, calibrated against non-mutant ATP1B2. Given is the mean +/- SD of 3 (patch II region 4 T240A and S) or 4 (other patch II mutants) independent experiments. Neg. control: untransfected cells.

X-fold mutant type	ATP1B2 patch II variant	MFI (% of non-mutant ATP1B2)
-	Neg. control	31.1 +/- 6.4
Not mutated	ATP1B2	100 +/- 0.0
Single mutants	Patch II region 1	137.6 +/- 37.7
	Patch II region 2	30.4 +/- 3.2
	Patch II region 3	112.6 +/- 36.6
	Patch II region 4 (T240L)	119.9 +/- 40.4
	Patch II region 4 (T240A)	62.4 +/- 29.5
	Patch II region 4 (T240S)	106.9 +/- 34.0
Double mutant	Patch II region 1+3	227.1 +/- 73.4

Importantly, the single-point mutant ATP1B2-T240L (ATP1B2 patch II, region 4 mutant) failed to bind retinoschisin, but was expressed at the cell surface. Moreover, the robust binding of the anti-ATP1B2 antibody in FACS analysis excludes profound structural alterations of this mutant protein. This suggest that T240 in patch II is crucial for retinoschisin binding by the Na/K-ATPase.

**Fig 6:**
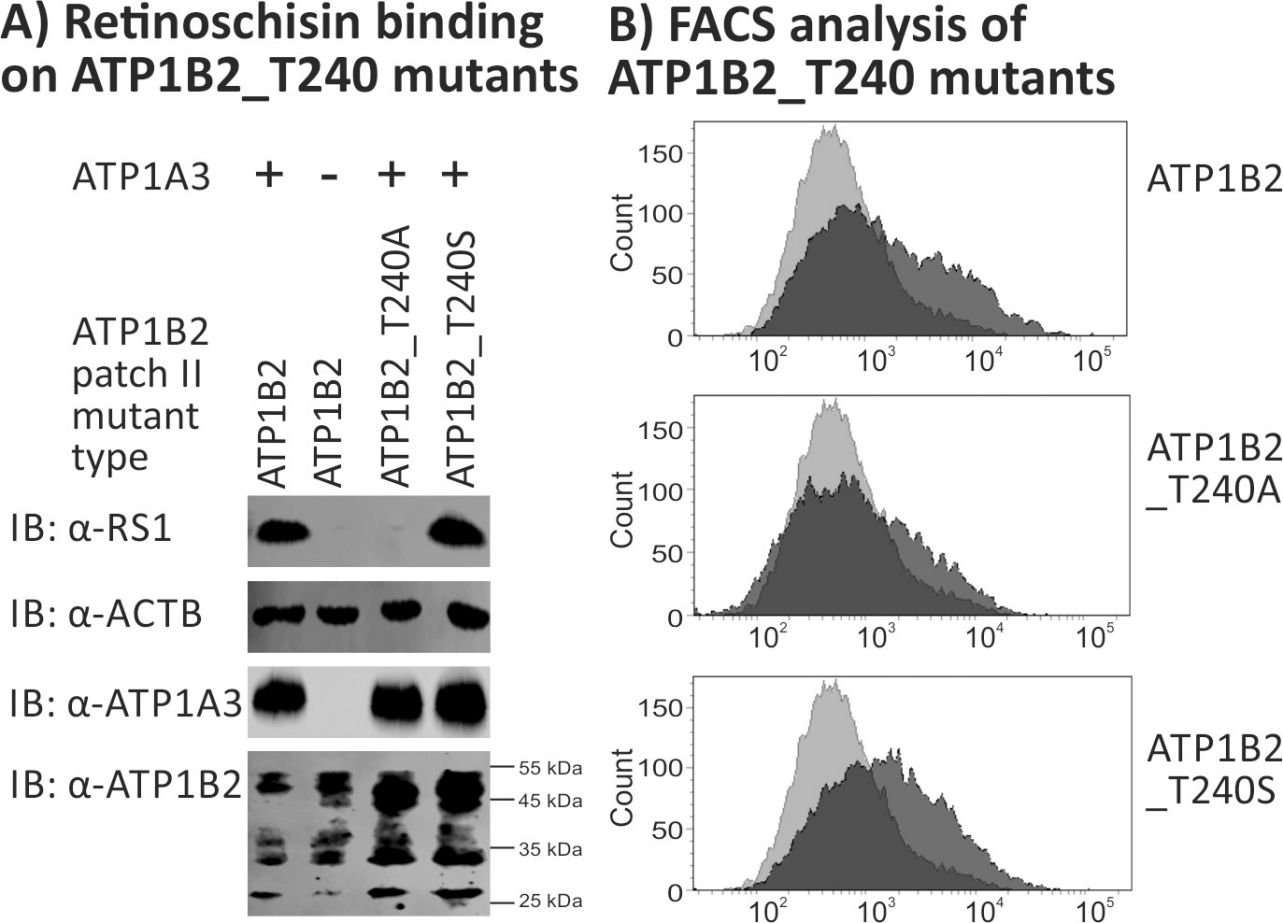
Retinoschisin binding to ATP1B2_T240A and ATP1B2_T240S. HEK293 cells were transfected with expression constructs for ATP1A3 in combination with expression constructs for ATP1B2_T240A and ATP1B2_T240S. **(A)** 48 h after transfection, cells were subjected to recombinant retinoschisin for 1 h, followed by intensive washing. Cells transfected with expression constructs for only ATP1B2 served as a negative control, cells transfected with expression constructs for ATP1A3 and normal ATP1B2 served as a positive control in the retinoschisin binding assay [13]. Heterologous protein expression as well as retinoschisin binding was investigated by Western blot analyses with antibodies against retinoschisin, ATP1A3, and ATP1B2. The ACTB staining served as loading control. **(B)** 48 h after transfection, cells were subjected to FACS analyses applying an anti-ATP1B2 antibody, representative histograms are given in this figure. Light grey: histogram of untransfected HEK293 cells depicting unspecific background signals. Dark grey: histogram of transfected HEK293 cells.

The aa exchange from threonine to leucine at position 240 replaced the rather small and polar threonine by the bulky and neutral isoleucine. To deepen our insight into the role of T240 in retinoschisin binding, two additional ATP1B2 mutants were generated: In mutant ATP1B2-T240A, T240 was replaced by the non-bulky, chemically inert alanine. In mutant ATP1B2-T240S, T240 was replaced by the chemically similar serine. Binding assays performed with these two ATP1B2 mutants revealed no binding of retinoschisin to ATP1B2_T240A, but to ATP1B2_T240S **(Fig 6A)**. Both mutants were robustly detected in FACS analyses (**Table 3, Fig 6B**), although with a slight decrease in mean fluorescence intensity for ATP1B2_T240A, comparable to the decrease seen for ATP1B2 patch I single mutants which still were able to bind retinoschisin.

Our experiments show that retinoschisin binding requires a polar aa residue (serine or threonine) at position 240, while a conversion to a neutral aa (leucine or alanine) does no longer allow for retinoschisin interaction. T240 (patch II region 4, labelled in orange in **Fig 3B**) is localized within a cavity formed by patch II, in close proximity to the α subunit (depicted as a dark grey cartoon in **Fig 3B**). The SASA of T240 in patch II is 13 Å^2^, indicating that this aa is partially buried [27].

## Discussion

We focused on identifying the specific substructure of Na/K-ATPase subunit ATP1B2 which mediates its interaction with retinoschisin. We found that retinoschisin binding to the retinal Na/K-ATPase was independent of ATP1B2 glycosylation, demonstrating that the high affinity of retinoschisin to sugars, as reported by Dyka and colleagues [7], is unrelated to its interaction with the Na/K-ATPase. *In silico* analyses to predict putative protein-protein interaction sites on ATP1B2 identified two hydrophobic surface patches I and II. Disruption of individual regions within these patches and functional analyses finally pointed to residue T240 of ATP1B2 as being responsible for mediating stable retinoschisin binding.

While several interaction partners, such as the retinal Na/K-ATPase [6], L-type voltage gated calcium channels [11], β2 laminin [10], anionic phospholipids [9], or galactose [7], have been suggested as interaction partners of retinoschisin, it was the retinal Na/K-ATPase, in specific its ATP1B2 subunit, which was shown to be required for anchoring retinoschisin to plasma membranes [13, 21]. In consequence, retinoschisin affects Na/K-ATPase localization in the retina and Na/K-ATPase associated intracellular signaling cascades [13, 21, 23]. Binding of retinoschisin to the retinal Na/K-ATPase and targeting the Na/K-ATPase to its correct retinal site is thus expected to play a major role in retinal integrity. To obtain insight into this interaction, we aimed to narrow the binding site from the retinal Na/K-ATPase to retinoschisin.

Initially, we investigated the possibility that retinoschisin, exhibiting a high affinity for galactose [7], may bind to ATP1B2 through its interaction with any of the eight potential glycoside side chains of ATP1B2. Adding external galactose and glucose indeed suppressed retinoschisin binding to the retinal Na/K-ATPase, however only after applying very high (0.75 M) and thus rather unphysiological concentrations. Also, as a vast number of membrane (bound) proteins are glycosylated (reviewed e.g. by [33]), the high specificity of retinoschisin to the retinal Na/K-ATPase and retinal membranes is likely not explained by a simple interaction to galactose or glucose. In our experiments, neither removal of individual glycoside side chains of ATP1B2 nor full enzymatic ATP1B2 deglycosylation negatively affected binding of retinoschisin to the retinal Na/K-ATPase, largely excluding ATP1B2 glycoside side chains as ligands for retinoschisin. Moreover, retinoschisin mutant RS1-R141H, showing a comparable galactose affinity as normal retinoschisin [7], was not able to bind ATP1B2 or retinal membranes. Taken together, our results indicate that the high affinity of retinoschisin variants to sugars is not linked to their capacity to interact with the retinal Na/K-ATPase or with retinal membranes. The inhibitory effect of solubilized galactose or glucose on binding of retinoschisin to the retinal Na/K-ATPase might be explained by a competition between sugars and ATP1B2 for the same binding region at retinoschisin. Alternatively, galactose/glucose binding to a different retinoschisin interaction site might induce structural alterations at retinoschisin disrupting its Na/K-ATPase binding site. The physiological relevance, if any, of retinoschisin binding to these sugars cannot be resolved based on the current results. Galactose affinity by retinoschisin might be required for an interaction of retinoschisin with other glycosylated proteins of the ECM or the plasma membrane, like β2 laminin [10], an ECM component, or L-voltage gated calcium channels [11, 12], found in the presynaptic terminal of photoreceptors and bipolar cells (reviewed in [34]). By interconnecting ECM or plasma membrane components, retinoschisin could be involved in stabilizing the structural and functional integrity of the retina, processes which are altered in a mouse model of retinoschisin deficiency [22]. However, galactose binding might also simply represent a physiologically not relevant, evolutionarily conserved property of discoidin domain containing proteins [7], many of which exhibit galactose binding [35]. Similarly, Dyka and colleagues reported binding of galactose by discoidin domain containing Factor Va, which was abolished by the presence of phosphatidylserine, the actual ligand for Factor V [7]. Finally, binding of sugars could represent a regulatory mechanism, altering the affinity of retinoschisin (or other discoidin domain containing proteins) to their interaction partners.

We also focused on the protein surface structure of the extracellular domain of ATP1B2 in order to gain insight into possible mechanisms mediating protein-protein interaction. Applying the *in silico* prediction tool PresCont, we identified two hydrophobic surface patches on ATP1B2, patch I and patch II. Site-directed mutagenesis of the each four hydrophobic regions forming patch I or patch II was performed to assess an interaction of retinoschisin with either of the two predicted patches. Individual disruption of all patch I regions (1, 2, 3 and 4) did not interfere with retinoschisin binding to ATP1B2, as is the case with combined disruption of regions 1+2+3, 1+4, and 2+4. Overall structural alterations in ATP1B2 mutants harboring several mutated patch I regions, as revealed in FACS analyses, might explain abolished retinoschisin binding by the other tested patch I mutants (combined disruption of regions 3+4, 1+2+4, 1+3+4, 2+3+4, and 1+2+3+4). Patch I is thus likely not the retinoschisin interaction site. It is interesting to note that in some cases (ATP1B2 patch I region 1+2+3, ATP1B2 patch I region 1+4), the evident structural alteration revealed by FACS did not interfere with retinoschisin binding. However, different mutations induce different structural alterations, which, as a consequence, can differently affect the accessibility of individual epitopes. The specific alterations induced by mutants ATP1B2 patch I region 1+2+3 and ATP1B2 patch I region 1+4 might thus disturb the antibody binding site but not the retinoschisin binding site. While rather not implicated in retinoschisin binding, patch I might be involved in other interaction processes. ATP1B2 was initially identified as an adhesion molecule on glia (AMOG) [36] and plays an important role in intercellular adhesion between neurons and astrocytes in the brain. Interaction mechanisms and interaction partners of ATP1B2 have not been identified yet, but the localization and the high PresCont scores suggest patch I as the adhesion interface.

Interestingly, a substitution of T240, one of the four regions forming patch II, by leucine (which is present in the corresponding aa position of the non-retinoschisin binding ATP1B1) interfered with retinoschisin binding but did not affect secretion or the overall surface structure of ATP1B2, as revealed by cell surface protein isolation and FACS analysis. To emphasize the role of T240 in retinoschisin binding, we additionally substituted T240 by the chemically inert alanine, an approach that is commonly used for analyzing protein-protein interfaces (e.g. [37–39]), again resulting in a mutant protein which was incapable of binding retinoschisin. Replacing T240 by the chemically similar serine, however, did not interfere with retinoschisin binding indicating that a hydroxyl moiety at aa 240 plays a key role in retinoschisin binding. A similar finding showing the necessity of a hydroxyl moiety at a defined aa position for ligand binding was reported for the human prostaglandin EP_2_ and EP_4_ receptors [40]. In specific, a threonine at position 185 or 168 of the EP_2_ and EP_4_ receptors, respectively, could be replaced by serine but not by alanine to still allow for ligand binding by the receptors. In agreement with these findings, bioinformatic approaches to detect critical aa residues in protein-protein binding sites observed that, although overall binding sites are hydrophobic, they harbor conserved polar residues at specific locations, serving as “hot spots” in the protein interfaces [41, 42]. Taken together, this suggest an involvement of patch II in retinoschisin binding, with a predominant role for residue T240.

3D modelling indicates that the binding-determining residue T240 is partially buried. In addition, due to its lateral position and proximity to ATP1A3, a direct interaction of retinoschisin with T240 (or A240) appears to require structural rearrangements of the Na/K-ATPase and thus an “induced fit mechanism of interaction” to improve accessibility of T240 (or S240). We can thus not exclude that T240 (or S240) is not directly involved in protein interaction, but rather renders the overall structure of patch II to accommodate retinoschisin binding. In support of this suggestion, structural rearrangements of the Na/K-ATPase enabling stable ligand binding are commonly observed upon interaction with other ligands like cardiac glycosides or ATP [43, 44].

The hyperflexibility of the suggested retinoschisin interaction domain, i.e. the spikes protruding from its discoidin domain [30, 45], should play an important role in mediating ATP1B2 binding. In 2007, Kiedzierska and colleagues [46] described a fundamental role of discoidin spikes in ligand binding revealing that hyperflexible discoidin spikes (regions protruding from the discoidin domain) intercalate into the interaction domains of their respective ligands. Data from cryo-electron microscopic analyses show that the retinoschisin spike regions face outward of the cogwheel-like retinoschisin octamer structure [45, 47]. Tolun and colleagues consequently suggested a lateral binding of interaction partners to the spikes, with the flat surface of the rings facing the membrane planes. In line with these results, functional and *in silico* analyses of secreted retinoschisin mutants imply a crucial role of the hyperflexible retinoschisin spike region for binding capacity and specificity [30]. Taken together, these studies suggest an induced fit mechanism of binding between retinoschisin and the retinal Na/K-ATPase, initiated by the intercalation of hyperflexible retinoschisin spikes into the ATP1B2 binding domain.

In summary, this study provides further insight into the binding mechanism of the retinal Na/K-ATPase with the retinoschisin protein. We identified a hydrophobic patch localized at the lateral site of the outer domain of ATP1B2 which likely represents the interface for retinoschisin. Importantly, residue T240 within this patch seems to play a crucial role in mediating stable binding to retinoschisin. Despite previous studies showing an interaction of retinoschisin to sugars, glycosylation of the retinal Na/K-ATPase appears not to play a role in retinoschisin binding.

## Supporting information

S1 Materials and Methods Statement on institutional animal care and use. (PDF)

S1 Table Oligonucleotides applied in expression cloning. (PDF)

S1 Fig Transfection efficiencies of ATP1B2 mutants. Densitometric quantification of ATP1B2 expression in **(A)** HEK293 cells co-transfected with expression vectors for ATP1A3 and for ATP1B2 glycosylation mutants (applied in retinoschisin binding assays, see Fig 2A, 3 independent replicates) **(B)** HEK293 cells co-transfected with expression vectors for ATP1A3 and for ATP1B2 patch I mutants (applied in retinoschisin binding assays, see Fig 4A, 3 independent replicates) **(C)** HEK293 cells co-transfected with expression vectors for ATP1A3 and for ATP1B2 patch II mutants (applied in retinoschisin binding assays, see Fig 5A, 3 independent replicates). **(D)** HEK293 cells co-transfected with expression vectors for ATP1A3 and for ATP1B2_T240 mutants (applied in retinoschisin binding assays, see Fig 6A, 3 independent replicates. No statistical significant difference was obtained in relative expression levels of the different ATP1B2 variants (p > 0.05). Expression levels did also not correlate with retinoschisin binding.
(PDF)

S2 Fig Binding of retinoschisin to HEK293 cells heterologously expressing the retinal Na/K-ATPase in the presence of sugars– 7 h incubation time with retinoschisin and sugars. HEK293 co-transfected with expression constructs for ATP1A3 and ATP1B2 for 48 h were subjected to recombinant retinoschisin for 7 h in the presence of 0 M (control) or 0.75 M galactose, glucose, or mannose, followed by intensive washing. Subsequently, the retinoschisin binding was analyzed *via* immunocytochemistry with antibodies against retinoschisin and ATP1B2. Scale bars, 40 μm.
(PDF)

S3 Fig Na/K-ATPase and retinal membrane binding of retinoschisin and RS1-R141H. (A) HEK293 cells co-transfected with expression constructs for ATP1A3 and for ATP1B2 for 48 h or enriched membranes of *Rs1h*^*-/Y*^ murine retinae were subjected to recombinant retinoschisin or retinoschisin mutant RS1-R141H for 1 h, followed by intensive washing. Cells transfected with expression constructs for only ATP1A3 or enriched membranes of *Rs1h*^*-/Y*^ murine kidney served as a negative control in the retinoschisin binding assay. Na/K-ATPase expression as well as retinoschisin or RS1-R141H binding was investigated by Western blot analyses with antibodies against retinoschisin, ATP1A3, ATP1B2, and ATP1B1. The ACTB staining served as loading control for HEK293. (B) HEK293 co-transfected with expression constructs for ATP1A3 and ATP1B2 for 48 h were subjected to recombinant retinoschisin or retinoschisin mutant RS1-R141H for 1 h, followed by intensive washing. Subsequently, the retinoschisin binding was analyzed *via* immunocytochemistry with antibodies against retinoschisin and ATP1B2. Scale bars, 20 μm. Despite a high affinity of both retinoschisin and RS1-R141H to immobilized sugars [7], only retinoschisin can bind to the retinal Na/K-ATPase heterologously expressed in HEK293 and to murine retinal membranes.
(PDF)
