# Supplementary material for: Identification of the retinoschisin-binding site on the retinal Na/K-ATPase
Source: PLoS One. 2019 May 2;14(5):e0216320. doi: 10.1371/journal.pone.0216320 (PMC6497308; doi:10.1371/journal.pone.0216320)
Supplement: S1 Materials and Methods — (PDF) [file pone.0216320.s001.pdf]

S1 Materials and Methods. Statement on institutional animal care and use.

Universität Regensburg

To Whom it May Concern

According to the *German Animal Welfare Act* (article 15) as well as to the *European Guidelines* (2010/63/EU) all animal experiments with vertebrates carried out in Germany have to be approved by the competent governmental authorities (*Regierung von Unterfranken, Wuerzburg, Germany*).

Each approved animal protocol gets a separate approval number. If vertebrates are euthanized without any pretreatment (e. g. to collect organs or tissues) there is no approval from the authorities needed.

At the **University of Regensburg** (*UR*) there is also an *Internal Animal Care and Use Committee* ("IACUC": "*Tierschutzausschuss*": Two scientists, two animal care takers, and the animal welfare officer) acting according to the *German Animal Welfare Act*.

Additionally to the governmental approval an *Institutional Review Board* (*IRB*: "*Kommission Tierlaboratorien*": Ten scientists from the life sciences, dean of the medical faculty, animal welfare officer, head of laboratory animal facilities) at the *UR* weighs the importance of every experimental procedure (including euthanasia) against the suffering inflicted to animals.

Three full-time *Veterinary Specialists in Laboratory Animal Science* are responsible for veterinary care. They have the full authority to make decisions about animal welfare (3R programs) and they are also responsible for hygiene programs (to maintain health and prevent disease), housing (including environmental enrichment), and management of all laboratory animal facilities at the *UR*, as well.

Every animal experiment (including euthanasia without pretreatment) is carried out under supervision of the *Animal Welfare Officer* of the *UR* who is to be contacted for further details.

Regensburg, September 3rd 2018

Th. Spruss

Thilo Spruss, Dr. rer. nat.

Animal Welfare Officer and Head of Laboratory Animal Facilities
Veterinary Specialist in Laboratory Animal Science
Veterinary Specialist in Animal Protection
Veterinary Specialist in Pharmacology & Toxicology
E-Mail: thilo.spruss@ur.de
Tel: +941 9446457
Fax: +941 9445373
http://www.uni-regensburg.de/forschung/zentrale-tierlaboratorien/index.html
