# Supplementary material for: Identification of the retinoschisin-binding site on the retinal Na/K-ATPase
Source: PLoS One. 2019 May 2;14(5):e0216320. doi: 10.1371/journal.pone.0216320 (PMC6497308; doi:10.1371/journal.pone.0216320)
Supplement: S1 Table — (PDF) [file pone.0216320.s002.pdf]

S1 Table. Oligonucleotides applied in expression cloning

Name	5'-3' Sequence	Application
hATP1B2-N96Q-F	GAG AAC CTT GAT GTC ATT GTC CAA GTC AGT GA	Expression cloning of ATP1B2 glycosylation mutants
hATP1B2-N96Q-R	TCA TGA CTT GGA CAA TGA CAT CAA GGT TCT C	
hATP1B2-N118Q-F	GGA GCC TTA CCA AGA CTC TAT CCA AGC CCA AAA G	
hATP1B2-N118Q-R	CTT TTG GGC TTG GAT AGA GTC TTG GTA AGG CTC C	
hATP1B2-N153Q-F	CAA ACG TGC CTG CCA ATTCCA ACG GAC CCA G	
hATP1B2-N153Q-R	CTG GGT CCG TTG GAA TTG GCA GGC ACG TTT G	
hATP1B2-N159Q-F	ACC CAG CTG GGC CAA TGC TCC GGC ATT	
hATP1B2-N159Q-R	AAT CCG GAG CAT TGG CCC AGC TGG GT	
hATP1B2-N193Q-F	CCG GGT CAT CAA CTT CTA TGC AGG AGC ACA ACA GAG CAT GAA TG	
hATP1B2-N193Q-R	CAT TCA TGC TCT GTT GTG CTC CTG CAT AGA AGT TGA TGA CCC GG	
hATP1B2-N197Q-F	CAA ACC AGA GCA TGC AAG TTA CCT GTG CTG	
hATP1B2-N197Q-R	CAG CAC AGG TAA CTT GCA TGC TCT GGT TTG	
hATP1B2-N238Q-F	AAA AGT TCC ACG TGC AAG ACA CAC AGC CC	
hATP1B2-N238Q-R	GGG CTG TGT GTC TTG CAC GTG GAA CTT TT	
hATP1B2-N250Q-F	CTG TGA AGT TCC TGC AAG TGA CCC CCA ACG	
hATP1B2-N250Q-R	CGT TGG GGG TCA CTT GCA GGA ACT TCA CAG	
RS1_R141H_F	ACC CAG GGG CAC TGT GAC ATC	Expression cloning of RS1-R141H
RS1_R141H_R	GAG GAT CCC TGA AAT CAC TTT G	
hATP1B2_PatchI_Reg1_F	CCG GCC TTG GGG ACT CCA CCC ACT ATG G	Expression cloning of ATP1B2 patch I mutants
hATP1B2_PatchI_Reg1_R	AGC AGT TGC CCA GCT GGG TCC GGT TGA A	
hATP1B2_PatchI_Reg2_F	TGT ACT GGG AAG CGA GAT GAA GAT GCTG	
hATP1B2_PatchI_Reg2_R	TTG AAC ATT CAT GCT CTG GTT TGC TCC T	
hATP1B2_PatchI_Reg3_F	TTC ACG AAT GTG ACC CCC AAC GTG GAG	
hATP1B2_PatchI_Reg3_R	CTG CACAGC CAC CAG GGG CTG TGT GTAG	
hATP1B2_PatchI_Reg4_R	AGT ACT CCA CGT TGC CGA GAT TCT CAG CAT CTT CAT CT	
hATP1B2-start_KpnI_F	CCC GGT ACC ATG GTC ATC CAG AAA GAG AAG	
hATP1B2_PatchI_Reg4_F	AGT ACT TCG GCC TGG GCA ACA GCC CCA ACA TCG ACC TCA TGT ACT TCC CCT ACT AT	
hATP1B2-stop_XhoI_R	CTC GAG GGT TTT GTT GAT GCG GAG TTT GA	

Name	5‘-3‘ Sequence	Application
hATP1B2_PatchII_Reg1_F	CCT CAG ATC GAG AAC CTT GAT GTC ATT GTC AAT GTC AGT G	Expression cloning of ATP1B2 patch II mutants
hATP1B2_PatchII_Reg1_R	AAT CTG TGT CAA GCC CGG TGT GGC CAG	
hATP1B2_PatchII_Reg2_1F	AGT TAG GTT CTT GGA GCC TTA CAA CG	
hATP1B2_PatchII_Reg2_1R	ATGTTC AGA ACA TGC TGG TCC CAG CT	
hATP1B2_PatchII_Reg2_2F	CAA AGA CTC TAT CCA AGC CCA AAA G	
hATP1B2_PatchII_Reg2_2R	CAA AGA CTC TGC CCA AGC CCA AAA G	
hATP1B2_PatchII_Reg2_3F	CAA AGA CTC TGC CCA AGC CCA AAA G	
hATP1B2_PatchII_Reg2_3R	TAC TTC TCC AAGAAC CTA AC	
hATP1B2_PatchII_Reg3_3F	AAC CGA GTC ATC AAC TTC TAT GCA G	
hATP1B2_PatchII_Reg3_3R	GAG CTT GAT GAA GAC ACA GGG CTG	
hATP1B2_PatchII_Reg4_F	CGT GAA CTA CCT GCA GCC CCT GG	
hATP1B2_PatchII_Reg4_R	TGG AAC TTT TTG CCA TAG	
hATP1B2_T240A_F	CGT GAA CTA C GC ACA GCC CCT GG	
hATP1B2_T240A_R	TGG AAC TTT TTG CCA TAG TAG GGG	
hATP1B2_T240S_F	CGT GAA CTA CTC ACA GCC CCT GG	
hATP1B2_T240S_R	TGGAACTTTTTGCCATAGTAGGG	
hATP1B2-KpnI-F	GGTACCATGGTCATCCAGAAAGAGAAGAA	Expression cloning of ATP1B2 and ATP1A3 for biscistronic vector
hATP1B2-XhoI-R	CTC GAG TCA GGT TTT GTT GAT GCG GAG T	
hATP1A3-NotI-F	GCG GCC GCA TGG GGG ACA AGA AAG ATG ACA A	
hATP1A3-BamHI-R	GGA TCC CTG GCT GAC GGG AAT GTT G	
