# Supplementary material for: Identification of the retinoschisin-binding site on the retinal Na/K-ATPase
Source: PLoS One. 2019 May 2;14(5):e0216320. doi: 10.1371/journal.pone.0216320 (PMC6497308; doi:10.1371/journal.pone.0216320)
Supplement: S1 Fig — Densitometric quantification of ATP1B2 expression in (A) HEK293 cells co-transfected with expression vectors for ATP1A3 and for ATP1B2 glycosylation mutants (applied in retinoschisin binding assays, see Fig 2A, 3 independent replicates) (B) HEK293 cells co-transfected with expression vectors for ATP1A3 and for ATP1B2 patch I mutants (applied in retinoschisin binding assays, see Fig 4A, 3 independent replicates) (C) HEK293 cells co-transfected with expression vectors for ATP1A3 and for ATP1B2 patch II mutants (applied in retinoschisin binding assays, see Fig 5A, 3 independent replicates). (D) HEK293 cells co-transfected with expression vectors for ATP1A3 and for ATP1B2_T240 mutants (applied in retinoschisin binding assays, see Fig 6A, 3 independent replicates. No statistical significant difference was obtained in relative expression levels of the different ATP1B2 variants (p > 0.05). Expression levels did also not correlate with retinoschisin binding. (PDF) [file pone.0216320.s003.pdf]

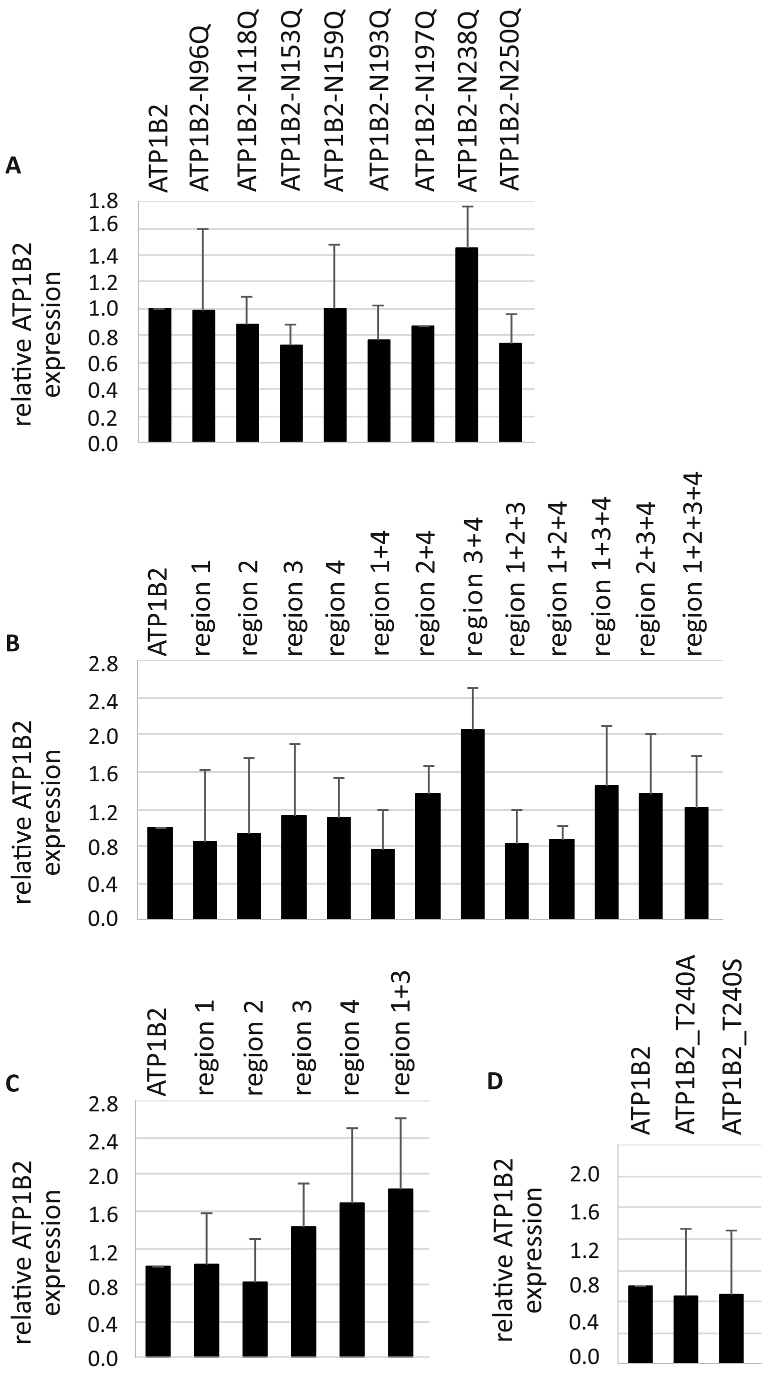

S1 Fig. **Transfection efficiencies of ATP1B2 mutants.** Densitometric quantification of ATP1B2 expression in **(A)** HEK293 cells co-transfected with expression vectors for ATP1A3 and for ATP1B2 glycosylation mutants (applied in retinoschisin binding assays, see Fig 2A, 3 independent replicates) **(B)** HEK293 cells co-transfected with expression vectors for ATP1A3 and for ATP1B2 patch I mutants (applied in retinoschisin binding assays, see Fig 4A, 3 independent replicates) **(C)** HEK293 cells co-transfected with expression vectors for ATP1A3 and for ATP1B2 patch II mutants (applied in retinoschisin binding assays, see Fig 5A, 3 independent replicates). **(D)** HEK293 cells co-transfected with expression vectors for ATP1A3 and for ATP1B2_T240 mutants (applied in retinoschisin binding assays, see Fig 6A, 3 independent replicates). No statistical significant difference was obtained in relative expression levels of the different ATP1B2 variants ($p > 0.05$). Expression levels did also not correlate with retinoschisin binding.
