# Supplementary material for: Identification of the retinoschisin-binding site on the retinal Na/K-ATPase
Source: PLoS One. 2019 May 2;14(5):e0216320. doi: 10.1371/journal.pone.0216320 (PMC6497308; doi:10.1371/journal.pone.0216320)
Supplement: S2 Fig — HEK293 co-transfected with expression constructs for ATP1A3 and ATP1B2 for 48 h were subjected to recombinant retinoschisin for 7 h in the presence of 0 M (control) or 0.75 M galactose, glucose, or mannose, followed by intensive washing. Subsequently, the retinoschisin binding was analyzed via immunocytochemistry with antibodies against retinoschisin and ATP1B2. Scale bars, 40 μm. (PDF) [file pone.0216320.s004.pdf]

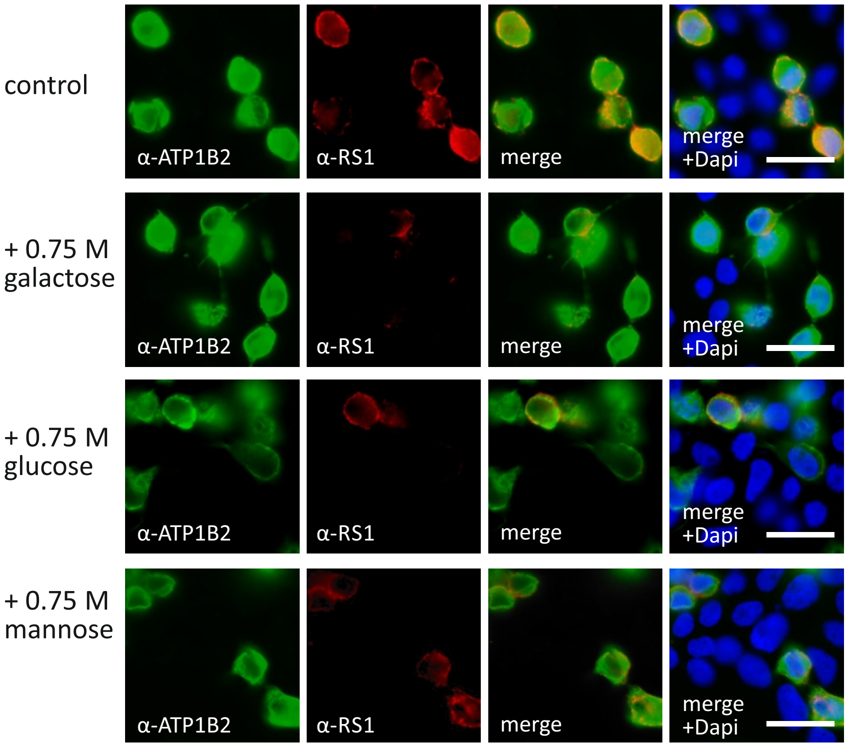

S2 Fig. Binding of retinoschisin to HEK293 cells heterologously expressing the retinal Na/K-ATPase in the presence of sugars – 7h incubation time with retinoschisin and sugars. HEK293 co-transfected with expression constructs for ATP1A3 and ATP1B2 for 48 h were subjected to recombinant retinoschisin for 7 h in the presence of 0 M (control) or 0.75 M glucose, galactose, or mannose, followed by intensive washing. Subsequently, the retinoschisin binding was analyzed *via* immunocytochemistry with antibodies against retinoschisin and ATP1B2. Scale bars, 40 µm.
