# Supplementary material for: Identification of the retinoschisin-binding site on the retinal Na/K-ATPase
Source: PLoS One. 2019 May 2;14(5):e0216320. doi: 10.1371/journal.pone.0216320 (PMC6497308; doi:10.1371/journal.pone.0216320)
Supplement: S3 Fig — (A) HEK293 cells co-transfected with expression constructs for ATP1A3 and for ATP1B2 for 48 h or enriched membranes of Rs1h-/Y murine retinae were subjected to recombinant retinoschisin or retinoschisin mutant RS1-R141H for 1 h, followed by intensive washing. Cells transfected with expression constructs for only ATP1A3 or enriched membranes of Rs1h-/Y murine kidney served as a negative control in the retinoschisin binding assay. Na/K-ATPase expression as well as retinoschisin or RS1-R141H binding was investigated by Western blot analyses with antibodies against retinoschisin, ATP1A3, ATP1B2, and ATP1B1. The ACTB staining served as loading control for HEK293. (B) HEK293 co-transfected with expression constructs for ATP1A3 and ATP1B2 for 48 h were subjected to recombinant retinoschisin or retinoschisin mutant RS1-R141H for 1 h, followed by intensive washing. Subsequently, the retinoschisin binding was analyzed via immunocytochemistry with antibodies against retinoschisin and ATP1B2. Scale bars, 20 μm. Despite a high affinity of both retinoschisin and RS1-R141H to immobilized sugars [7], only retinoschisin can bind to the retinal Na/K-ATPase heterologously expressed in HEK293 and to murine retinal membranes. (PDF) [file pone.0216320.s005.pdf]

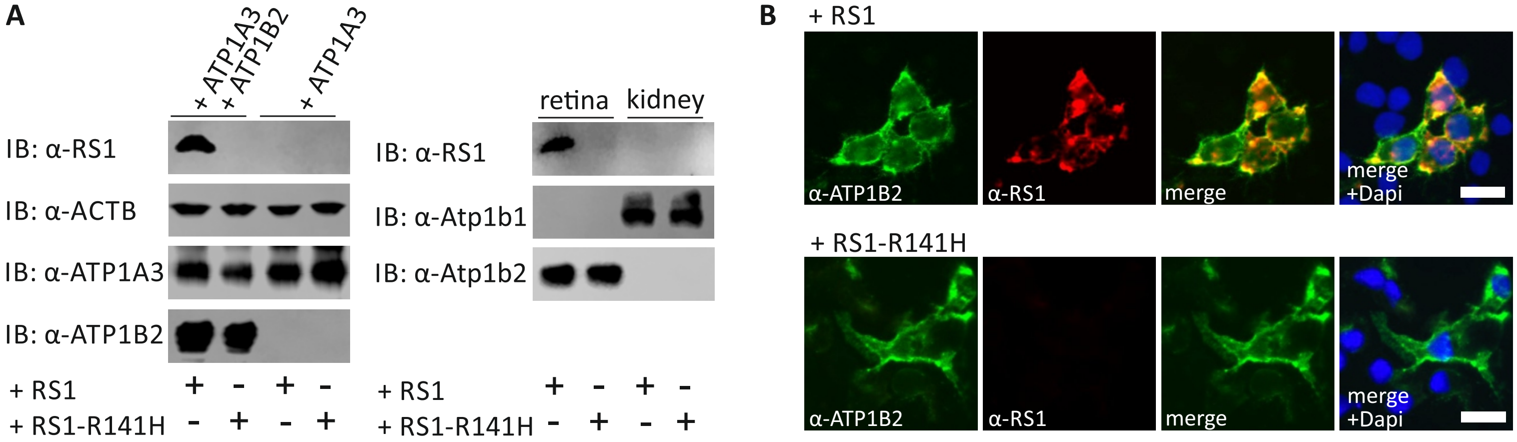

S3 Fig. Na/K-ATPase and retinal membrane binding of retinoschisin and RS1-R141H. (A) HEK293 cells co-transfected with expression constructs for ATP1A3 and for ATP1B2 for 48 h or enriched membranes of *Rs1h*$^{-/Y}$ murine retinae were subjected to recombinant retinoschisin or retinoschisin mutant RS1-R141H for 1 h, followed by intensive washing. Cells transfected with expression constructs for only ATP1A3 or enriched membranes of *Rs1h*$^{-/Y}$ murine kidney served as a negative control in the retinoschisin binding assay [13]. Na/K-ATPase expression as well as retinoschisin or RS1-R141H binding was investigated by Western blot analyses with antibodies against retinoschisin, ATP1A3, ATP1B2, and ATP1B1. The ACTB staining served as loading control for HEK293. **(B)** HEK293 co-transfected with expression constructs for ATP1A3 and ATP1B2 for 48 h were subjected to recombinant retinoschisin or retinoschisin mutant RS1-R141H for 1 h, followed by intensive washing. Subsequently, the retinoschisin binding was analyzed *via* immunocytochemistry with antibodies against retinoschisin and ATP1B2. Scale bars, 20 µm. Despite a high affinity of both retinoschisin and RS1-R141H to immobilized sugars [7], only retinoschisin can bind to the retinal Na/K-ATPase heterologously expressed in HEK293 and to murine retinal membranes.
